# Sequential Pattern Mining to Predict Medical In-Hospital Mortality from Administrative Data: Application to Acute Coronary Syndrome

**DOI:** 10.1155/2021/5531807

**Published:** 2021-05-25

**Authors:** Jessica Pinaire, Etienne Chabert, Jérôme Azé, Sandra Bringay, Paul Landais

**Affiliations:** ^1^UPRES EA 2415-Clinical Research University Institute, Montpellier University, Montpellier 34 093, France; ^2^LIRMM-UMR 5506, Montpellier University, Montpellier 34 093, France; ^3^AMIS, Paul Valéry University, Montpellier 34 199, France

## Abstract

Prediction of a medical outcome based on a trajectory of care has generated a lot of interest in medical research. In sequence prediction modeling, models based on machine learning (ML) techniques have proven their efficiency compared to other models. In addition, reducing model complexity is a challenge. Solutions have been proposed by introducing pattern mining techniques. Based on these results, we developed a new method to extract sets of relevant event sequences for medical events' prediction, applied to predict the risk of in-hospital mortality in acute coronary syndrome (ACS). From the French Hospital Discharge Database, we mined sequential patterns. They were further integrated into several predictive models using a text string distance to measure the similarity between patients' patterns of care. We computed combinations of similarity measurements and ML models commonly used. A Support Vector Machine model coupled with edit-based distance appeared as the most effective model. We obtained good results in terms of discrimination with the receiver operating characteristic curve scores ranging from 0.71 to 0.99 with a good overall accuracy. We demonstrated the interest of sequential patterns for event prediction. This could be a first step to a decision-support tool for the prevention of in-hospital death by ACS.

## 1. Introduction

Prediction of a medical outcome based on a trajectory of care has generated a lot of interest in medical research [1]. International experience shows that the spectrum of application is wide: preventive medicine, improving care and quality of life, and reducing healthcare costs [2, 3]. Furthermore, transition to electronic healthcare systems has led to the accumulation of vast amounts of medical data. Healthcare data is becoming just as important as administrative data, genomic, medical. As a result, medical data mining has great potential for exploring hidden patterns in vast medical datasets [2]. For healthcare management, data mining prediction appears to be a promising tool [4].

With about 17.5 million deaths a year, cardiovascular diseases represent the first leading cause of death in the world [5]. Future projections anticipate that the number of fatalities will reach 24 million in 2030 and these disorders will remain the leading cause of mortality. In France, about 120,000 people are affected each year: 12,000 die during the first episode and 18,000 in the following year. Furthermore, cardiovascular diseases play an important role in healthcare consumption, and this leads to the most substantial expense of medical goods and services. In France, cardiovascular diseases accounted for 0.8% of the gross domestic product which represented 15.6 billion euros in 2014 [6]. As the population grows older, these expenditures are expected to increase considerably [7]. In this context, one of the main issues is to predict acute coronary syndrome (ACS) risk of mortality.

As sequence prediction has many application domains (web page prefetching, product recommendation, stock market prediction, weather forecasting, and sequence prediction of clinical events), various models have been developed based on machine learning methods (Markov models, directed graphs, neural networks models) [8–13], grammar inference [14, 15], or process mining [16, 17]. Review of the literature showed that methods based on machine learning techniques outperform other models [18]. In addition, an important issue associated with sequence prediction is to reduce model complexity. To address this challenge, a number of solutions have been proposed including combination of pattern mining techniques with pattern matching techniques [11]. Based on these findings, this paper investigates such techniques for sequence prediction.

In a previous work, we highlighted the interest of patient trajectories as a decision tool [19]. In this article, our objective is to show that this tool can be useful in predicting hospital mortality. The originality of our work is to use patient trajectories as predictors through similarity scores, while considering the medical particularity of each type of population. We suggested an innovative ACS in-hospital death modeling protocol based on patient care history from administrative databases. Then, sequential patterns were integrated in the model by using a similarity distance. We considered the most common predictive models currently available, combined with text string measures, in order to uncover the most suitable combination *(model, similarity)*. Our prediction protocol is based on the TRIPOD guidelines [20]. We applied this method to the French Hospital Discharge Database (FHDD). We compared this method to the most popular current predictive models.

Our contribution relates to methodological aspects and medical implications. We detailed the technique used, and then we applied this approach to the morbid events chronology to predict in-hospital mortality after an ACS. Then, we discussed the results and options for generalizing this methodology according to the data typology together with the retained data mining approach; we also suggested applications for medical practice.

## 2. Materials and Methods

### 2.1. Dataset

Every year, all French public or private health facilities caring for medical and surgical patients submit de-identified patients' data to the FHDD [21]. Each discharge summary submitted to the FHDD is linked to a national grouping algorithm leading to a French Diagnosis Related Group (DRG) [22]. This study was conducted according to the approval given by the Commission Nationale de l'Informatique et des Libertés (CNIL), agreement No. 1375062. The present dataset of ACS has been collected from the FHDD for the 2009–2014 period. Patients with an ACS were extracted according to the following International Classification of Diseases 10th revision (ICD-10) codes: I21 to I24 and the percutaneous coronary intervention codes (see Table S1 in the Supplementary Material). A previous work presented the global database [23].

#### 2.1.1. Inclusion Criteria

We focused on the French metropolitan population >45 years old. In addition, we included patients who experienced at least four stays related to cardiovascular diseases. Finally, we included 4,871 patients in the analysis, in whom 668 in-hospital deaths occurred.

#### 2.1.2. Sequential Database

For each patient, a sequence of DRGs and a sequence of ICD-10 codes were identified. Their lengths were equal to the number of patients' stays over the six-year observation period. We performed a filtering process to remove irrelevant stays, i.e., stays that were unrelated to cardiovascular diseases. These filtered sequences of DRGs, and ICD-10 codes were called patient trajectories.

#### 2.1.3. Contextual Information

**Figure 1:**
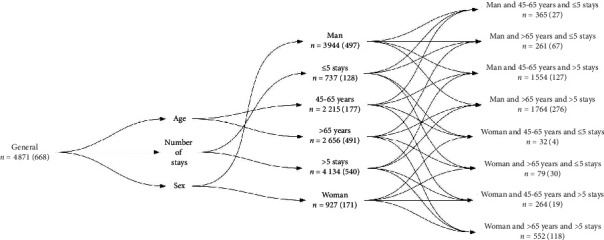
The contextual hierarchy. Number (*n*) of patients is displayed with number of deaths in parentheses.

To consider the differences (age, sex, comorbidities, etc.) in cardiovascular diseases, the dataset was divided into sub-populations, also called contexts, by using covariates like sex, age, and number of hospitalizations [23]. To preserve medical consistency, quantitative variables were discretized. Two classes of age have been defined: 45–65 years and >65 years. In the 45–65 age group (45% of the population), incipient coronary heart disease might lead to ACS. In the >65 age group (55% of the population) several risk factors, accrued with aging (diabetes, dyslipidemia, hypertension, etc.), increase the risk of ACS. The average number of hospitalizations was 5. For the sake of simplification, we illustrated our procedure for two classes: ≤5 stays and >5 stays.  Figure 1 presents the hierarchy of contexts.

## 3. Methods

**Figure 2:**
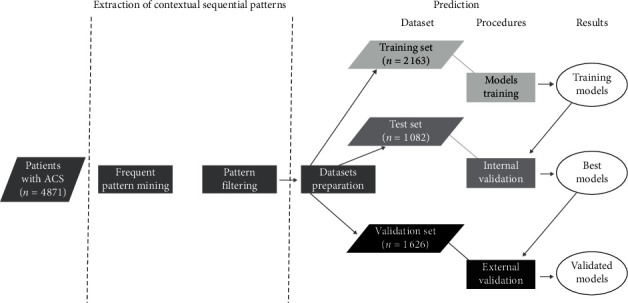
Data flow chart.

Figure 2 presents our data flow chart. Two modules were implemented: (1) extraction of contextual sequential patterns and then (2) prediction by context.

### 3.1. Extraction of Contextual Sequential Patterns

In this first module, we extracted frequent care path profiles in patient trajectories considering contextual information frequently associated with sequential data. We proceeded in two steps:

#### 3.1.1. Frequent Pattern Mining

Table 1 presents an example. Each patient has a list of time-ordered events corresponding to ICD-10 codes (R07: pain in throat and chest; I20: angina pectoris; I25: chronic ischemic heart disease; I21: acute myocardial infarction (AMI); I50: heart failure). As an example, for *P*_1_ patient, diagnoses R07 and I21 appeared in February, then diagnosis I20 in April. These events are called *items*. An *itemsetit*_*i*_ is a non-ordered group of events occurring at the same time. A *sequenceS*=*it*_*a*_, *it*_*b*_,…, *it*_*p*_ is a non-empty and ordered list of *p* itemsets. Sequences are associated with a context (e.g., a man >65 years).

A pattern is supported by a patient if this pattern is included in its sequence of events. For instance, the pattern *P*=(R07)(I20) is included in the sequence of *P*_1_ to *P*_5_  patients (Table 1). The support of a *P* pattern in a *c*_*i*_ context, denoted by support_*c*_*i*__(*P*), is defined as the percentage of patients supporting *P* in  *c*_*i*_. Let *c*_1_={>65 years} and *c*_2_={45 − 65 years} 45–65 years then support_*c*_1__(*P*)=(8/9)  and support_*c*_2__(*P*)=(1/6). A threshold, called *minimum support*, is required to find the most frequent patterns: the extracted patterns must have a support greater than this threshold. Let the minimum support be equal to 2/3; *P* is frequent in *c*_1_ but not in *c*_2_. We mined contextual frequent patterns in ACS trajectories using an efficient algorithm, called CFPM (Contextual Frequent Pattern Mining) [24], based on the *PrefixSpan* algorithm [25].

#### 3.1.2. Pattern Filtering

To avoid redundant information increasing risk to introduce collinearity in predictive models, we filtered the results of step (a). We retained the maximal frequent patterns [26]. Let *F*={(I20), (I20)(I21)(I25), (I20)(I21), (I21)(I25), (I21)(I20), (I20)(I21)(I24)}, be the set of frequent patterns in ICD-10 code sequences; in a given *c* context, this set becomes *F*′={(I20)(I21)(I25), (I21)(I20), (I20)(I21)(I24)}.

Finally, we obtained a list of the maximum frequent patterns by context.

### 3.2. Prediction

In this module, we determined the best modeling and associated predictive performance. The code can be loaded at https://gite.lirmm.fr/advanse/myoctus.

The objective was to predict the binary event: alive or dead in a care facility. Based on the TRIPOD guidelines [20], it included four steps.

#### 3.2.1. Dataset Preparation

We randomly split the dataset into two parts: a first part (*n* = 3245) for training models and internal validation, and a second part (*n* = 1626) was kept for external validation. We made balanced samples, training (*n* = 2163) and test (*n* = 1082) sets. Balancing was completed on the variable to predict as many dead patients as living patients. Two contexts with low numbers (woman and 45–65 years and >5 stays and woman and 45–65 years and ≤5 stays) were not used in the model because information was insufficient to make accurate predictions.

We integrated patterns discovered in the previous module as predictors by measuring the similarity between these patterns and patients' trajectory. In a *c* given context, let *s*_1_^*c*^,…, *s*_*k*_^*c*^ be the *k* sequential patterns of the context. Let *P* a patient of the *c* context having a *T*_*P*_ trajectory. Then, sim_*P*_^*c*^, the *k*-length vector determining similarity between every pattern of *c* and *T*_*P*_,  is given by sim_*P*_^*c*^=(sim(*T*_*P*_, *s*_1_^*c*^),…, sim(*T*_*P*_, *s*_*k*_^*c*^)), where sim is a similarity measure between two strings. Here, calculating similarity is analogous to measuring the gap between two strings. There are three ways of comparing text string measures: edit-based distances, distances based on counting q-grams, and heuristic distances. We integrated the similarity measure in the model choice. We calculated similarities for the following distances: longest common substring distance, Levenshtein distance, optimal string alignment distance, Damerau-Levenshtein distance, q-gram distance, Jaccard distance, cosine distance, Jaro distance, and Jaro-Winkler distance [27].

#### 3.2.2. Training Models

Predictors were sex, age group, and similarities. The latter were discretized: low similarity (sim(*T*_*P*_, *s*_*i*_^*c*^) < 0.4); medium similarity (0.4 ≤ sim(*T*_*P*_, *s*_*i*_^*c*^) < 0.6); and strong similarity (sim(*T*_*P*_, *s*_*i*_^*c*^) ≥ 0.6). So, we integrated two kinds of variables: continuous or discretized similarities. Based on a cross-validation principle with training and test sets, we compared most popular models: Naïve Bayes (NB), k-nearest neighbors algorithm (KNN), regression tree (Tree), logistic regression (LR), support vector machine (SVM), and artificial neural networks (ANN) [28, 29].

#### 3.2.3. Internal Validation

We assessed the quality of the prediction by calculating the discrimination with the following criteria: accuracy, sensibility, specificity, error rate, precision, F-measure, and area under ROC curve (AURC). Based on these discrimination measures, we chose the *(model, similarity)* combination presenting the best compromise using the maximal vector computation method [30].

#### 3.2.4. External Validation

We evaluated the discrimination power and overall accuracy of selected models. Discrimination was assessed by AURC and overall accuracy by Brier Score [31].

## 4. Results

### 4.1. Extraction of Contextual Sequential Pattern Mining

We mined sequential patterns in DRG (and ICD-10 code) trajectories. As patterns will be used as predictors in the models, the longer they are, the more informative they will be and the better the prediction should be. We experimented with several supports in the sequential patterns' extraction. Most patients' trajectories were of a short length; besides, there was a great variability in their trajectories. Consequently, we reduced the support until 1% to extract a maximum of relevant patterns for predictive models. Most patterns mined were 1-item or 2-item sequential patterns (Table 2 illustrates two examples). We can observe that features are different according to sub-populations and consistent with the medical knowledge of the cardiovascular diseases.

### 4.2. Prediction

The prediction module evaluated six models, nine similarity measures, and two types of variables (discretized and continuous), i.e., 108 different models. We present grouped results by (1) class of models and similarities, or by (2)-(3) context.

#### 4.2.1. Best Combination of (Model, Similarity)

Internal validation used several key metrics.  Table 3 summarizes results for three key metrics (AURC, F-measure, and error rate) grouped by categories of models and similarities in all contexts. According to the key metric chosen, the selected combination may be different. According to the AURC criterion, the best combination was (SVM, heuristic). Considering the F-measure criterion, the best combination was (ANN, heuristic). Considering the error rate, the best combinations were (SVM, edition) and (ANN, edition). Overall, KNN models presented the worst performances according to the aforementioned criteria.

Table 4 presents the best combinations (in bold) resulting from the selection process. In most contexts, the best combinations were (SVM, edition) whatever the type of trajectory used. In addition, models with continuous similarities performed better than those with discretized similarities. We found height combinations with heuristic similarities associated with SVM, ANN, and LR models, essentially in the ≤5 stays group contexts. We also found six combinations with q-gram similarities that performed better.

Then, we focused on the models only. We aggregated the results by context, for each type of model, and ranked the models according to their performance.  Table 5 presents the results of this ranking in percentage for the three best performances (first to third). For ICD-10 code trajectory modeling (ICD-TM), SVM is the most efficient model in 74% of cases; then, at the second place, we found the ANN models (70%) and LR models at the third place (43%). For the DRG trajectory modeling (DRG-TM), we found ANN models mostly in first place (57%), but also in second place with SVM models (35%) and LR models in third place (35%).

#### 4.2.2. Advice on Figures

We explored the performances of the models defined above (see Table 6). AURC ranged from 0.71 to 0.99 for DRG-TM and ICD-TM. According to this criterion, the best results were found in the following contexts: man and 45–65 years and ≤5 stays, 45–65 years, and ≤5 stays for DRG-TM, and in woman and 45–65 years, ≤5 stays and also man and 45–65 years and ≤5 stays contexts for ICD-TM. Conversely, the worst models concerned the >65 years and > 5 stay's contexts in both DRG-TM and ICD-TM.

Considering the error rate criterion, if we focused on the contexts highlighted by the AURC criterion, we observed good results with rates reaching 2% and 3% depending on the type of trajectory modeling. However, best results were established for 45–65 years and ≤5-stay's context in DRG-TM (3%) and man and ≤5-stay's context in the ICD-TM (2%). Less efficient results were established for the >65 years and >5-stay's context in DRG-TM (32%) and for the 45–65 years and >5-stay's context in ICD-TM (39%).

We also observed good performances with high AURC values (upper than 0.8) associated with low error rates (less than 10%) in most of the contexts with the ≤5 stays category considering the two types of modeling (see Table 6). Conversely, poorer performances were observed in the contexts with the >5 stays category (error rate>20%). Furthermore, ≤5 stays contexts were smaller than >5 stays contexts since there were fewer patients in these contexts.

#### 4.2.3. External Validation

In the final step, we proceeded to an external validation (see Table 7). AURC varied from 0.65 to 0.99 in DRG-TM and from 0.57 to 0.91 in ICD-TM. The higher AURC values were found in the following contexts: man and 45–65 years and ≤5 stays for DRG-TM and 45–65 years and ≤5 stays for ICD-TM. Less discriminant case concerned the context of woman and 45–65 years for DRG-TM and the context of 45–65 years and >5 stays for ICD-TM.

In parallel, Brier Score ranged from 0.02 to 0.24 (0.09 to 0.26, resp.) in DRG-TM (ICD-TM, resp.). According to this criterion, the best overall accuracy was found in man and 45–65 years and ≤5 stays for DRG-TM and in >65 years and ≤5 stays for ICD-TM. In contrast, the worst overall accuracy concerned the context of man for DRG-TM and 45–65 years and >5 stays for the ICD-TM.

**Figure 3:**
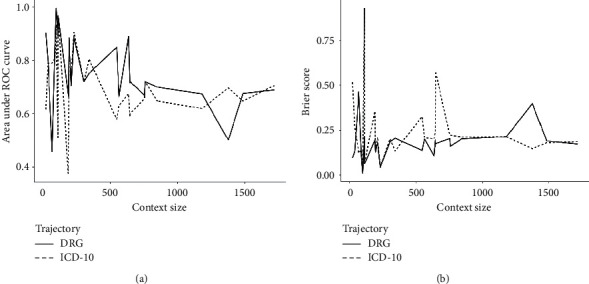
External validation: area under ROC curve and Brier Score according to the type of trajectory and context size. (a) Area under ROC curve. (b) Brier Score.

We compared AURC and Brier Score by type of modeling according to the number of patients belonging to the context (here for the validation set) or the context size in Figure 3. We obtained close validation results regardless of the given criterion for the two types of modeling. Comparing types of modeling, Figure 3 shows that results were better in the DRG-TM, except for several contexts: man and >65 years and ≤5 stays, ≤5 stays, 45–65 years, man, woman, and general. With the AURC, curves were close for both small and high context sizes. On the contrary, with the Brier Score, the curves were close for several intervals of context size. Link between validation sample size and performances in external validation was not obvious.

## 5. Discussion

### 5.1. Best Combinations of (Model, Similarity)

In most contexts, SVM model coupled with edit-based distance was the most efficient combination associated with in-hospital mortality. However, in most of cases, ANN models were the second effective model followed by LR models. These three types of models have quite equivalent performances in terms of calibration and discrimination. In other investigations, similar results were raised. For instance, LR models provide satisfactory results in predicting in-hospital mortality in patients with AMI [32]. In addition, comparing ANN, SVM, and LR models for mortality prediction in patients with cardiovascular diseases, differences were not significant between machine learning models and classical regression models [33–35]. Some researchers have shown that the decision trees outperformed the LR, ANN, and SVM algorithms in mortality prediction but they used intensive care unit data [36]. Furthermore, a review, examining risk prediction models with electronic health records data, reported that linear regression models were the most common algorithms used with high level of accuracy [37].

Comparing the combinations of *(model, similarity)* performances by model, combinations with edit-based distance were often the most efficient. In string distances, the choice usually depends on the nature of the data and the length of the sequences. For example, q-gram distances are well suited for very long length sequences contrarily to heuristic distances [38, 39]. Besides, we observed a q-gram distance associated with the model essentially for the contexts including the >5 stays category and/or the >65 years age group. In the latter, length trajectories were substantially longer. Indeed, older people have more medical events as a result of ageing than younger age groups. Similarly, in the >5 stays context concerning patients with comorbidity, sequences were longer. Conversely, a heuristic distance appeared most frequently for contexts including ≤5 stays category and/or the 45–65 years category where trajectories were potentially shorter associated with younger age. Thus, our results were consistent with the choice of the final distance selection as well as the length of the sequences.

Furthermore, models with discretized similarities had worse results than those with continuous. Indeed, dichotomization of continuous variables leads to a loss of information. Thus, this was consistent with the conclusions of a previous work [40].

### 5.2. Modeling Competitiveness

A comparison study evaluating established risk prediction models for cardiovascular disease showed that performances varied from 0.71 to 0.88 according to the AURC criterion [41]. Here, AURC ranged from 0.71 to 0.99. Thus, our models were competitive compared to state-of-the-art models in dealing with ACS mortality risk prediction model. Besides, we had the best performances in small size contexts. Indeed, in these contexts sampling allowed covering more diverse situations.

However, we might refine our results by improving different steps of our protocol. Firstly, the contextual sequential patterns mining module might be improved by (a) creating different contexts by using comorbidities or the type of care procedure performed to mine patterns that would be more specific to a sub-population and (b) combining DRG and ICD-10 code sequences or even adding information such as related diagnoses, care procedures, or even comorbidity scores like Charlson or Elixhauser [42]. Hence, we might extract patterns from sequences of several itemsets instead of one as in our approach. Secondly, the prediction module might be developed using other approaches: (a) competing other models like random forest, boosted trees or classifications and regression trees; (b) selecting features with techniques like wrappers, filters, or embedded methods [43]; (c) tuning the final models while adjusting their parameters with optimization algorithms [44].

### 5.3. Limits

Our study has several limitations impacting the modeling results.

#### 5.3.1. Duration of the Study Observation

Most trajectories were of a short length; thus many patterns were of a short length, which impacted model efficiency. Some investigations, based on cardiovascular registries, examining hospitalization trends in ACS have a history of up to twenty-five years [45]. At that time, the French PPS did not go back that far. By contrast, the one used by the French health insurance system is longer. However, in the latter database only the follow-up care and drug prescriptions are compiled but without diagnoses or comorbidities that did not enable a similar analysis to the one we present.

#### 5.3.2. Time Gaps in Sequential Patterns

In our modeling, we did not consider the time gap between events. However, this could be an important information in this type of modeling because time-lapse between successive hospitalizations in the patient trajectory can vary from days to months and even years. An extension to this work is possible using survival models [46], with the process described in the article by using mining time-gap sequential patterns models [47]. Other approaches were developed based on machine learning methods. For example, an association of methods based on a combination of embedding entities and events in a multidimensional latent space with a Markov model to predict the sequence of events recorded in the electronic medical record of each patient has given promising results [48]. More recently, two methods for time-dependent event representation have been proposed combined with a RNN model [49]. These methods offer new perspectives when the number of instances is important.

#### 5.3.3. Complementary Covariates

We did not consider the influence of comorbid conditions. It might be of interest to integrate comorbidity index that summarizes disease burden and can discriminate for in-hospital mortality, such as the Charlson or Elixhauser score [42] in the modeling [50].

#### 5.3.4. The Choice of the Database: The NHDDB

In the case of these diseases, treatment involves hospitalization and hospital follow-up. Thus, our study resumes most of the patients and important events. However, for ease of modeling, we considered that the patient was still alive if the death was not observed during the hospital stays. Access to the national cause of death statistics set up by the CépiDC (INSERM) would remedy this [51].

#### 5.3.5. Model Power

In our population, in-hospital mortality accounted for 14% of all patients. But, analyzing population by context, this proportion ranged from 7% to 38% for man and 45–65 years and ≤5 stays, and woman and >65 years and ≤5 stays, respectively. As we made balanced samples, with rare events, we lost information, especially in large size contexts. Consequently, the power of our models was low. Other approaches for predicting rare events [52] might be more appropriate for some contexts with a low proportion of deaths. In this instance, simulation studies would be of interest, increasing the number of events by resampling, in order to increase the power of the models [53, 54].

### 5.4. Beyond the Technique: Implications for Medical Practice

Predicting the next event in a patient's care pathway is important information that could help to estimate the benefit of a care strategy and its cost-effectiveness. In addition, understanding the risk factors for ACS mortality provides clinicians and patients with important information to guide both prognosis and appropriate treatment [55]. Thus, the approach presented here is a first step towards a more elaborate approach with multiple implications in medical practice. To illustrate our point, we proposed some examples from the literature.

A next step in the development of a prognostic model based on the medico-administrative database might be its implementation directly in the health system where the data are routinely collected. Thus, this device would provide performance measures of care units by health facilities and would allow comparison between them across the national territory [56]. Indeed, this hospital mortality monitoring tool for ACS takes all the more sense in the implementation of the Territory Hospitals Group (created by The 2009 French Law on “Hospitals, Patients, Health, and Territories” and the 2016 “Health System Modernization Act”) [57, 58], one of whose objectives is to ensure equal access to safe and quality of care throughout the country [59]. Additional work on medical procedures and comorbidity scores could lead to adjustment models depending on the patient's medical context. This would improve performance measures and enhance the quality care delivered to the population.

Other monitoring applications might be elaborated by building scoring tools. For example, it is possible to compare medical procedures by estimating a risk score to predict in-hospital mortality associated with percutaneous coronary catheterization or surgery [60, 61]. Hence, this approach would be an aid to identify patients most likely to be affected by adverse outcomes and consequently be oriented towards treatment alternatives. For example, the GRACE risk prediction model provides rapid and widely applicable method for assessing cardiovascular risk that can guide patient triage and management across the spectrum of patients with ACS [62].

Our approach can be generalized in a more generic approach to predict the next medical event. Thus, enriching the model with complementary data [63] such as diagnoses, medical procedures, laboratory results [64], or comorbidity scores [50], would allow assessing the risk of a second cardiac event for patients with recent myocardial infarction [65]. Therefore, this will help to identify patients most likely to benefit from secondary prevention therapies. In addition, preventing relapses participates in the improvement of care resources distribution and consequently contributes to reducing related health costs.

Finally, a last example of application in ACS may be the identification of risk factors contributing to ACS mortality burden [66]. To achieve this objective, improvements have been proposed previously, on the one hand by enriching the models with complementary features, and on the other hand by using resampling techniques to increase the power of the models. This knowledge of risk factors and in particular prognostic trajectories could enable caregivers to inform patients but also to adapt their care strategy [67, 68]. This could then contribute to reducing ACS mortality which is a crucial public health issue.

There are also many examples of application considering other conditions than ACS, to show the value to exploit the information contained in the patient trajectories in a predictive achievement, as an example, the prediction of risk mortality in intensive care units (ICU) which is an important issue. Indeed, FHDD contains a lot of data and more precisely key indicators related to health status measured timely during the hospitalization period in ICU. Since short-term mortality is rare even in patients with severe disease, it is difficult to predict. However, a recent study shows that clinical data trajectories is an efficient feature in a well-designed machine learning model to assess the risk of mortality and can also be helpful to address this last issue [69].

To go further with these illustrations, we can focus on a patient's general health condition. All medical visits and health interventions are recorded in electronic medical records. These data represent the patient's medical history, but also might contain information on an emerging trajectory of an illness leading to a major issue and/or requiring specific medical intervention. Based on this hypothesis, a deep learning approach has been developed to predict future medical events [70]. This approach is called DeepCare and showed its efficacy for disease progression modeling, intervention recommendation, and future risk prediction.

These examples are concrete cases and illustrate the various utilities of using patient trajectories in a prediction goal: health planning, cost reduction, prediction of major health issue, or comorbid conditions [71], highlighting paths leading to a specific medical event.

## 6. Conclusion

We used sequential patterns to elaborate in-hospital mortality prognostic models. Sequential patterns were integrated as predictors by measuring a similarity between patients' trajectory and patterns. We ensured competition between the most popular string distances of the literature. We built our prediction protocol, using the TRIPOD guidelines. We used the most commonly predictive models for comparison. Our purpose was to establish the best *(model, similarity)* combination by context.

SVM model coupled with an edit-based distance with similarity as a continuous variable was the combination that offered the most efficient performances. Other perspectives of comparisons are conceivable using survival models like Cox model [46] or even predictive models based on sequences [72]. In a future work, we plan to mine sequential patterns in a sequence of itemsets combining DRG, ICD-10, medical procedure, and related diagnosis codes. This modeling could provide a better understanding of the interrelationships between care pathways and associated diseases that increase the risk of death.

This first approach, presenting encouraging results, with further developments might have several applications for medical practice. First, with a monitoring tool, it might contribute to measure the burden of ACS, but also to improve healthcare [56]. Second, with a risk score, it might provide an aid for the patient triage and facilitate the patient care by providing a decision-support tool to orient towards the most convenient care strategy for instance [65]. To conclude, better knowledge of the relationship between care pathways, associated with comorbidities and mortality, might help to combat this public health issue by reducing the ACS mortality burden [66]. We limited our study to hospitalization data; however an extension of this work could be carried out using the data and causes of death extracted from the French Health Data Hub (Système National des Données de Santé: SNDS) allowing adding important elements such as prescription of drugs, deaths outside establishment hospital, and consultations with a cardiologist, outside the hospital, or patient monitoring [73].

## Figures and Tables

**Table 1:** Example of patients' sequential database.

Patient	Age (years)	Sex	January	February	March	April
*P*_1_	>65	Man		**R07**, I21		**I20**
*P*_2_	>65	Man	**R07**, I50		**I20**	
*P*_3_	>65	Man	**R07**	R07		**I20**
*P*_4_	>65	Man	I25	**R07**		**I20**, I25
*P*_5_	>65	Man	I21	**R07**, I25	**I20**, I25, I21	
*P*_6_	>65	Woman		I20		R07
*P*_7_	>65	Woman		**R07**	**I20**	R07
*P*_8_	>65	Woman	I21	**R07**		**I20**, I25
*P*_9_	45–65	Man		**R07**, R07	R07	**I20**, I21
*P*_10_	45–65	Man			I20, I25, I21	
*P*_11_	45–65	Man			I20, I21	R07
*P*_12_	45–65	Woman	I50	I20, I25, I21		R07
*P*_13_	45–65	Woman		I20, I21, I50		
*P*_14_	45–65	Woman	I20		R07	I50

The <(R07) (I20)> pattern appears in bold with contextual information on sex and age. This pattern appeared in individuals aged >65 years. Only one 45–65 years individual was included.

**Table 2:** Two examples of the most frequently mined contextual sequential patterns in ACS trajectories together with their corresponding support.

Sequential pattern	Support
*Man and 45–65 years and >5 stays*	
<(Chronic ischemic heart disease)>	42.4
<(Angina pectoris)>	32.6
<(AMI)>	29.5
<(Angina pectoris) (angina pectoris)>	6.1
<(Angina pectoris) (chronic ischemic heart disease)>	4.4
<(Chronic ischemic heart disease) (chronic ischemic heart disease) (chronic ischemic heart disease)>	1.8

*Woman and >65 years and ≤5 stays*	
<(AMI)>	45.4
<(Angina pectoris)>	25.2
<(Chronic ischemic heart disease)>	24.5
<(Chronic ischemic heart disease) (chronic ischemic heart disease)>	2.7
<(AMI) (chronic ischemic heart disease)>	1.9
<(AMI) (AMI)>	1.9

**Table 3:** Means of area under the ROC curve (AURC), F-measure, and error rate for the different types of models and similarities in the modeling of ICD-10 code trajectories.

		AURC		F-measure		Error rate	
Model	Similarity	Mean	95% CI	Mean	95% CI	Mean	95% CI
NB	Edition	0.77	0.68–0.86	0.70	0.62–0.82	0.26	0.16–0.34
	q-gram	0.72	0.64–0.77	0.64	0.58–0.70	0.33	0.28–0.38
	Heuristic	0.73	0.64–0.82	0.66	0.60–0.77	0.32	0.24–0.39

KNN	Edition	0.44	0.38–0.53	0.58	0.53–0.63	0.38	0.35–0.43
	q-gram	0.50	0.45–0.55	0.57	0.52–0.61	0.40	0.37–0.44
	Heuristic	0.54	0.46–0.59	0.55	0.52–0.65	0.41	0.38–0.46

Tree	Edition	0.74	0.66–0.83	0.66	0.56–0.79	0.28	0.19–0.35
	q-gram	0.67	0.62–0.71	0.63	0.57–0.70	0.34	0.30–0.39
	Heuristic	0.70	0.64–0.80	0.65	0.57–0.77	0.31	0.22–0.38

LR	Edition	0.77	0.68–0.88	0.70	0.62–0.83	0.27	0.16–0.35
	q-gram	0.75	0.65–0.82	0.69	0.62–0.77	0.29	0.23–0.38
	Heuristic	0.74	0.64–0.82	0.69	0.62–0.80	0.30	0.21–0.39

SVM	Edition	0.83	0.76–0.92	0.70	0.61–0.82	**0.25**	0.16–0.33
	q-gram	0.80	0.72–0.89	0.66	0.60–0.73	0.31	0.26–0.37
	Heuristic	**0.84**	0.77–0.92	0.70	0.64–0.81	0.27	0.20–0.36

ANN	Edition	0.82	0.72–0.94	0.70	0.59–0.85	**0.25**	0.14–0.33
	q-gram	0.81	0.72–0.90	0.70	0.62–0.79	0.28	0.21–0.37
	Heuristic	0.83	0.71–0.96	**0.73**	0.63–0.86	0.26	0.14–0.35

CI = confidence interval. Best results are in bold.

**Table 4:** Distribution (%) of the best combinations (model, similarity) according to the type of trajectories.

	ICD-10 code trajectories				DRG trajectories			
	Tree	LR	SVM	ANN	Tree	LR	SVM	ANN
Edition	—	2.86	**42.86**	17.14	5.56	—	**53.70**	20.37
q-gram	—	5.71	5.71	2.86	—	—	—	1.85
Heuristic	5.71	—	11.43	5.71	—	3.70	11.11	3.70

**Table 5:** Average ranking (%) of the best models across all contexts and similarities.

Rank	ICD-10 code trajectories						DRG trajectories					
	NB	KNN	Tree	LR	SVM	ANN	NB	KNN	Tree	LR	SVM	ANN
1st	—	—	—	4.35	73.91	21.74	—	—	—	17.39	34.78	56.54
2nd	4.35	—	4.35	4.35	17.39	69.57	—	—	8.70	21.74	34.78	34.78
3rd	30.43	—	13.04	43.48	8.70	4.35	30.43	—	13.04	26.09	21.74	4.35

**Table 6:** Internal validation: AURC, error rate, numbers of predicted, and observed deaths by context according to the type of trajectory.

Context	DRG trajectories				ICD-10 code trajectories			
	AURC	Error rate	Observed	Predicted	AURC	Error rate	Observed	Predicted
Man and >65 years and ≤5 stays	0.93	0.11	16	12.6	0.98	0.08	16	14
Woman and 45–65 years	0.96	0.05	15	14.8	**0.99**	0.07	15	13.4
>65 years and ≤5 stays	0.91	0.09	16	14.6	0.85	0.14	16	13.4
Man and 45–65 years and ≤5 stays	**0.99**	0.04	16	15.4	**0.99**	0.05	16	15.6
45–65 years and ≤5 stays	**0.99**	**0.03**	16	15.2	0.98	0.05	16	15.6
Woman and >65 years and >5 stays	0.87	0.22	20	16.8	0.93	0.2	20	18.4
Woman and >65 years	0.92	0.16	26	22.6	0.87	0.18	26	24.6
Man and ≤5 stays	0.97	0.07	14	14	0.97	**0.02**	14	13.8
≤5 stays	0.97	**0.03**	20	19	**0.99**	0.04	20	19
Woman and >5 stays	0.93	0.19	24	16.8	0.96	0.20	24	20
Woman	0.93	0.13	30	24.2	0.94	0.14	30	26.8
Man and 45–65 years and >5 stays	0.89	0.27	22	20.4	0.86	0.27	22	17.2
Man and >65 years and >5 stays	0.82	0.29	46	32	0.84	0.28	46	34.2
45–65 years and >5 stays	0.86	0.27	24	19.8	0.78	0.39	24	16.2
Man and 45–65 years	0.93	0.07	15	14.2	0.78	0.35	26	20.4
Man and >65 years	0.84	0.25	56	42.4	0.80	0.28	56	44.2
45–65 years	0.82	0.26	30	22	0.82	0.29	30	18.6
>65 years and >5 stays	0.71	0.32	66	31.6	0.71	0.33	66	63.6
>65 years	0.76	0.26	82	56.2	0.74	0.27	82	48.4
Man and >5 stays	0.79	0.31	70	56.4	0.74	0.33	70	59.2
Man	0.78	0.30	84	94.6	0.92	0.23	84	79.4
>5 stays	0.82	0.28	92	88	0.82	0.26	92	75.2
General	0.81	0.25	114	69.4	0.81	0.25	114	88.6

Best results are in bold.

**Table 7:** External validation: AURC and Brier Score by context according to the type of trajectory.

Context	DRG trajectories		ICD-10 code trajectories	
	AURC	Brier Score	AURC	Brier Score
Man and >65 years and ≤5 stays	0.87	0.11	0.82	0.15
Woman and 45–65 years	0.65	0.12	0.65	0.11
>65 years and ≤5 stays	0.90	0.09	0.88	**0.09**
Man and 45–65 years and ≤5 stays	**0.99**	**0.02**	0.81	0.11
45–65 years and ≤5 stays	0.96	0.05	**0.91**	0.14
Woman and >65 years and >5 stays	0.77	0.19	0.74	0.19
Woman and >65 years	0.75	0.14	0.81	0.16
Man and ≤5 stays	0.95	0.08	0.89	0.14
≤5 stays	0.94	0.05	0.87	0.16
Woman and >5 stays	0.67	0.21	0.79	0.20
Woman	0.83	0.13	0.82	0.16
Man and 45–65 years and >5 stays	0.80	0.18	0.65	0.24
45–65 years and >5 stays	0.78	0.18	0.57	0.26
Man and >65 years and >5 stays	0.68	0.23	0.75	0.19
Man and 45–65 years	0.80	0.14	0.76	0.24
Man and >65 years	0.76	0.14	0.80	0.18
45–65 years	0.74	0.22	0.81	0.17
>65 years and >5 stays	0.70	0.17	0.70	0.24
>65 years	0.79	0.15	0.72	0.17
Man and >5 stays	0.77	0.20	0.74	0.21
Man	0.76	0.24	0.85	0.17
>5 stays	0.73	0.21	0.80	0.19
General	0.82	0.16	0.79	0.17

Best results are in bold.

## Data Availability

All relevant data are within the manuscript and its supporting information files. The original data source is not accessible because it is protected by data confidentiality. The data are stored by a third party, which delivers the permission to access these data in the same manner as the authors. The request for data must be sent to the Système National des Données de Santé (SNDS). The procedure is clearly described at https://www.snds.gouv.fr/SNDS/Processus-d-acces-aux-donnees'.

## Supplementary Materials

Supplementary Materials Table S1. List of the percutaneous coronary interventions used by the identification algorithm of acute coronary syndrome from the FHDD.
