# Supplementary material for: Sequential Pattern Mining to Predict Medical In-Hospital Mortality from Administrative Data: Application to Acute Coronary Syndrome
Source: J Healthc Eng. 2021 May 25;2021:5531807. doi: 10.1155/2021/5531807 (PMC8172301; doi:10.1155/2021/5531807)
Supplement: Supplementary Materials — Table S1. List of the percutaneous coronary interventions used by the identification algorithm of acute coronary syndrome from the FHDD. [file 5531807.f1.docx]

## Supplementary Materials

Table A.1. List of the percutaneous coronary interventions used by the identification algorithm of acute coronary syndrome from the FHDD.

| **Code** | **Detail** |
| --- | --- |
| DDAF001 | Intraluminal dilatation of a coronary vessel without stenting, transcutaneous arterial |
| DDAF003 | Intraluminal dilatation of 3 or more coronary vessels with stenting, transcutaneous arterial |
| DDAF004 | Intraluminal dilatation of 2 coronary vessels with stenting, transcutaneous arterial |
| DDAF006 | Intraluminal dilatation of a coronary vessel with stenting, transcutaneous arterial |
| DDAF007 | Intraluminal dilatation of 2 coronary vessels with coronary arteriography with stenting, transcutaneous arterial |
| DDAF008 | Intraluminal dilatation of a coronary vessel with coronary arteriography with stenting, transcutaneous arterial |
| DDAF009 | Intraluminal dilatation of 3 or more coronary vessels with coronary arteriography with stenting, transcutaneous arterial |
| DDAF010 | Intraluminal dilatation of a coronary vessel with coronary arteriography without stenting, transcutaneous arterial |
| DDFF001 | Intraluminal coronary artery atherectomy by rotational method, transcutaneous arterial |
| DDFF002 | Intraluminal coronary artery atherectomy by directional method, transcutaneous arterial |
